# PerBrain: a multimodal approach to personalized tracking of evolving state-of-consciousness in brain-injured patients: protocol of an international, multicentric, observational study

**DOI:** 10.1186/s12883-022-02958-x

**Published:** 2022-12-09

**Authors:** L. Willacker, T. M. Raiser, M. Bassi, A. Bender, A. Comanducci, M. Rosanova, N. Sobel, A. Arzi, L. Belloli, S. Casarotto, M. Colombo, C. C. Derchi, E. Fló Rama, E. Grill, M. Hohl, K. Kuehlmeyer, D. Manasova, M. J. Rosenfelder, C. Valota, J. D. Sitt

**Affiliations:** 1 Department of Neurology, University Hospital of the Ludwig-Maximilians-Universität München, Marchioninistr. 15, Munich, Germany; 2 Department of Biomedical and Clinical Sciences, University of Milano, Milan, Italy; 3 Therapiezentrum Burgau, Hospital for Neurological Rehabilitation, Burgau, Germany; 4 IRCCS Fondazione Don Carlo Gnocchi ONLUS, Milan, Italy; 5 Department of Brain Sciences, Weizmann Institute of Science, Rehovot, Israel; 6 Sorbonne Université, Institut du Cerveau - Paris Brain Institute - ICM, Inserm, CNRS, 75013 Paris, France; 7 Department of Medical Neurobiology and Department of Cognitive and Brain Sciences, The Hebrew University of Jerusalem, Jerusalem, Israel; 8 Laboratorio de Inteligencia Artificial Aplicada, Instituto de Ciencias de la Computación, Universidad de Buenos Aires, Buenos Aires, Argentina; 9 Consejo Nacional de Investigaciones Científicas y Técnicas (CONICET), Ministry of Science, Technology and Innovation, Buenos Aires, Argentina; 10 Institute for Medical Information Processing, Biometry and Epidemiology, Ludwig-Maximilians-Universität München, Munich, Germany; 11 German Center for Vertigo and Balance Disorders, Klinikum der Universität München, Munich, Germany; 12 Institute of Ethics, History and Theory of Medicine, Ludwig-Maximilians-Universität München, Munich, Germany; 13 Université Paris Cité, Paris, France; 14 Clinical and Biological Psychology, Institute of Psychology and Education, Ulm University, Ulm, Germany

**Keywords:** Disorders of consciousness, Multimodal neurodiagnostic, Outcome prediction, Caregiver well-being, Machine learning, Multicentric evaluation, Family caregivers, Personalized medicine

## Abstract

**Background:**

Disorders of consciousness (DoC) are severe neurological conditions in which consciousness is impaired to various degrees. They are caused by injury or malfunction of neural systems regulating arousal and awareness. Over the last decades, major efforts in improving and individualizing diagnostic and prognostic accuracy for patients affected by DoC have been made, mainly focusing on introducing multimodal assessments to complement behavioral examination. The present EU-funded multicentric research project “PerBrain” is aimed at developing an individualized diagnostic hierarchical pathway guided by both behavior and multimodal neurodiagnostics for DoC patients.

**Methods:**

In this project, each enrolled patient undergoes repetitive behavioral, clinical, and neurodiagnostic assessments according to a patient-tailored multi-layer workflow. Multimodal diagnostic acquisitions using state-of-the-art techniques at different stages of the patients’ clinical evolution are performed. The techniques applied comprise well-established behavioral scales, innovative neurophysiological techniques (such as quantitative electroencephalography and transcranial magnetic stimulation combined with electroencephalography), structural and resting-state functional magnetic resonance imaging, and measurements of physiological activity (i.e. nasal airflow respiration). In addition, the well-being and treatment decision attitudes of patients’ informal caregivers (primarily family members) are investigated. Patient and caregiver assessments are performed at multiple time points within one year after acquired brain injury, starting at the acute disease phase.

**Discussion:**

Accurate classification and outcome prediction of DoC are of crucial importance for affected patients as well as their caregivers, as individual rehabilitation strategies and treatment decisions are critically dependent on the latter. The PerBrain project aims at optimizing individual DoC diagnosis and accuracy of outcome prediction by integrating data from the suggested multimodal examination methods into a personalized hierarchical diagnosis and prognosis procedure. Using the parallel tracking of both patients’ neurological status and their caregivers’ mental situation, well-being, and treatment decision attitudes from the acute to the chronic phase of the disease and across different countries, this project aims at significantly contributing to the current clinical routine of DoC patients and their family members.

**Trial registration:**

ClinicalTrials.gov, NCT04798456. Registered 15 March 2021 – Retrospectively registered.

## Background and objectives

### Background

Severe brain injuries following traumatic or non-traumatic etiologies can lead to Disorders of Consciousness (DoC), a class of neurological conditions characterized by pathological alterations of arousal and/or awareness. DoC patients are highly heterogeneous regarding type, extent, and location of the underlying structural and functional brain pathology as well as in terms of their clinical phenotype [1]. The DoC range from coma (patients’ eyes closed) to unresponsive wakefulness syndrome (UWS; eyes open either passively or in response to stimulation) - also known as vegetative state (VS) - to minimally conscious state (MCS; divided into “MCS-“: visual pursuit oberservable without signs of command following, and “MCS+”: command following without effective communication) [2, 3].

The diagnostic and prognostic tests available for DoC in the current clinical practice demonstrate an unacceptably high misdiagnosis rate of approximately 40% [4]. Although diagnostic accuracy can be improved by the application of well-established standardized behavioral rating scales such as the Coma Recovery Scale-Revised (CRS-R) [5], these scales still lack optimal sensitivity (as they require the preservation of patient’s sensory, motor and executive functions) [6, 7], and specificity (due to the lack of a ground-truth about consciousness in many patients with DoC) [8]. Hence, behavioural assessments may fail to detect consciousness in DoC patients due to injury-induced individual cognitive, sensory or motor deficits. Meta-analyses suggest that up to 15–20% of DoC patients are likely to exhibit covert signs of consciousness or cognitive-motor dissociation (CMD) [9], while seeming unresponsive purely based on behavioral bedside examinations [10, 11].

Current guidelines on the diagnosis of DoC recommend multimodal evaluations using a combination of standardized neurological examinations, (functional) neuroimaging, and neurophysiological tools in order to improve and individualize diagnostic and prognostic accuracy [12]. In particular, using innovative functional neurodiagnostic methods, which do not require behavioral command following of the examined patient, covert markers of consciousness can be detected and, in turn, diagnostic accuracy can be increased [13, 14]. Among these recommended methods, quantitative electroencephalography (qEEG), functional magnetic resonance imaging (fMRI), and EEG combined with transcranial magnetic stimulation (TMS-EEG) have shown promising results [10, 12, 15, 16]. Combining evidence obtained from different neurodiagnostic multimodal techniques and analyzing them with modern machine learning-based methods can further increase diagnostic accuracy [14, 17].

However, despite major progress in accurate diagnosis and outcome prediction for DoC patients over the last years [10], as to date, reliable prognostic indices and read-outs to prognosticate a patient’s clinical evolution are still missing [18]. Moreover, the etiology and pathophysiology of DoC are highly heterogeneous [19], and most likely result from the combination of several factors, whose role and interplay still need to be clarified. Similarly, the precise neuronal mechanisms underpinning consciousness and its loss and recovery are still poorly understood, especially at the individual patient level. However, for ethical, therapeutic, and economic reasons, it is imperative to improve diagnostic accuracy and to predict outcome as early, reliably, and accurately as possible [20–22]. Indeed, many medical decisions and further treatment or rehabilitation paths crucially rely on accurate diagnostic and prognostic results [23, 24].

In addition, more reliable diagnosis and prognosis are of utmost relevance not only for patients’ management but also for the patients’ family members who very often become their informal caregivers. Taking care of DoC patients is a highly stressful experience for relatives and can represent a great emotional burden for them. Informal caregivers are exposed to substantial life changes and consequently often report low mental and physical health as well as high levels of distress [25]. These burdens are related to the ambiguity of the situation they are facing, and especially to the uncertainty of its duration and outcome [26, 27]. At the same time, they might need to act as surrogate decision-makers for their loved ones. Neuroimaging evidence of covert consciousness could confront them with ethical challenges [28]. For these reasons, the development of personalized tools to improve diagnosis and prognosis of DoC as well as their comprehensive communication are of utter importance for both patients and their family members.

### Aims

The overall goal of the multicenter European PerBrain project (full project title: “PerBrain: A Multimodal Approach to Personalized Tracking of Evolving State-Of-Consciousness in Brain-Injured Patients”) is to optimize the diagnosis and long-term prognosis of DoC patients following acute brain injury by combining clinical and multimodal tools. The project aims at developing a hierarchical diagnosis and prognosis procedure that allows a personalized description of the neurological status and the expected recovery potential at the single patient level.

The hierarchically applied methods include a wide range of the state-of-the-art neuroimaging-based (structural MRI and fMRI) and neurophysiological-based (qEEG and TMS-EEG) techniques as well as the investigation of brain-body interactions (recordings of nasal respiration during rest and during odor presentation). Further, well-established standardized clinical behavioral scales for the assessment of consciousness are administered (CRS-R, Glasgow Outcome Scale Extended GOS-E). Data from the different investigation modalities will then integrated by employing newly developed biostatistical analysis based on machine learning with the aid of information technology. In this way, a better understanding of the pathophysiological mechanisms of DoC is expected to be gained, consequentially enabling more tailored rehabilitation strategies and improving prognosis at single patient level.

In parallel to the optimal definition of the patients’ neurological status, PerBrain aims at investigating factors that impact well-being of informal caregivers of DoC patients and their treatment decision attitudes, specifically when confronted with results of multimodal technology-based tests, with the goal to develop strategies for the effective communication of technology-based results specifically tailored to caregivers’ needs.

## Methods

### Study design and data collection

PerBrain is an international, multicentric, longitudinal mixed-methods project focusing on tracking the neurological status of DoC patients and the needs of their informal caregivers over the period of one year. It is coordinated by the Institut du Cerveau, Paris, and includes the following project partners: Department of Biomedical and Clinical Science of the University of Milano, Italy, IRCCS Santa Maria Nascente Fondazione Don Carlo Gnocchi ONLUS, Italy, Weizmann Institute of Science, Israel, University Hospital of the Ludwig-Maximilians-Universität München, Germany, and Therapiezentrum Burgau Hospital for Neurorehabilitation, Germany.

Each patient undergoes at least five standardized clinical examinations with the CRS-R within two weeks to establish a stable clinical diagnosis of consciousness before the first multimodal assessment takes place. Subsequently, in order to track the patient’s clinical evolution, neurophysiological assessments are performed at three time points within one year after injury: in the acute phase (T0; 1 - 2 months after injury), subacute phase (T1; 4 - 7 months after injury) and chronic phase (T3; 9 - 12 months after injury). The clinical and multimodal assessment includes 1. multiple behavioral evaluations using gold-standard scales (GOS-E and CRS-R), 2. neurophysiological evaluations based on qEEG (resting-state and task-based paradigms), and, where available, TMS-EEG, 3. neuroimaging evaluations based on structural MRI and resting-state fMRI, and 4. finally also repeated nasal airflow and respiration measurements (24 h rest recording and odor dependent). For a compilation of the study visits, see Table 1.

**Table 1:** Overview of patient (T1, T2, T3) and caregiver (C0, C1, C2, C3) assessments

C0 Before patients’ assessment	T1 1-2 m from injury	C1 1-2w after results communi-cation	T2 4-7 m from injury	C2 1-2w after results communi-cation	T3 9-12 m from injury	C3 1-2w after results communi-cation
Personal data FCV-19S Religious/ spiritual information BIPQ EQ-5D-5L AcQoL HADS RS14 SOC-13 Treatment choices survey	Patient’s multimodal assessment ^a^ + Doctor’s communication of results to caregivers	FCV-19S BIPQ EQ-5D-5L HADS + qualitative interview (N = 9)	Patient’s multimodal assessment ^a^ + Doctor’s communication of results to caregivers	FCV-19S BIPQ EQ-5D-5L AcQoL HADS Treatment choices survey	Patient’s assessment at home (CRS-R & GOS-E)	FCV-19S Religious/ spiritual information BIPQ EQ-5D-5L AcQoL HADS RS14 SOC-13

*m* months, *w* weeks, *FCV-19S* Fear of COVID-19 Scale; *BIPQ* Brief Illness Perception Questionnaire, *EQ-5D-5L* European Quality of Life-5 Dimensions, *AcQoL* Adult Carer Quality of Life Questionnaire, *HADS* Hospital Anxiety and Depression Scale, *RS14* 14-Item Resilience Scale, *SOC-13* 13-item Sense of Coherence Scale

^a^ Patient’s multimodal assessments at T1 and T2 include behavioral scales (CRS-R, GOS-E), qEEG, TMS-EEG, structural and functional MRI, and nasal respiration measurements (24h and odor dependent).

In case the patient’s legal surrogate gave informed written consent for the patient to take part in the research (patient study part), the patients’ family caregivers are asked to participate in the caregiver study part as well. For participating caregivers, quantitative and, for a subset of them, qualitative data concerning their psychological well-being, informational needs about their relative’s condition, and expectations are collected. For each included caregiver, four assessments are set (time points C0, C1, C2, and C3), comprising different subsets of questionnaires (see Table 2) which are in relation to the patients’ assessments (c.f. Table 1).

**Table 2:** Collected quantitative and qualitative caregiver data

Data	C0	C1	C2	C3
Sociodemographic characteristics	x			
Religious/spiritual information	x			x
FCV-19S	x	x	x	x
BIPQ (adapted to caregivers)	x	x	x	x
EQ-5D-5L	x	x	x	x
ACQoL	x		x	x
HADS	x	x	x	x
RS14	x			x
SOC-13	x			x
Treatment choices survey	x		x	
Qualitative interview		x		

Timepoints: C0 (before diagnostics), C1 (after T1, 1-2 months after onset of DoC), C2 (after T2, 4-7 months after onset of DoC), C3 (after T3, 9-12 months after onset of DoC)

Pseudonymised data from patients and caregivers as well as patients‘ metadata associated to their neuroimaging and physiological evaluations are collected and managed using a custom longitudinal REDCap data base [29, 30]. The database was structured in events (enrollment, clinical evaluation, EEG evaluation, nasal respiration measurements, TMS-EEG, MRI, caregiver appointments C0, C1, C2 and C3) and for each event specific forms were constructed to store relevant information of patients and their corresponding caregivers. The enrollment event contains forms related to demographics, injury etiology, patients’ medical history and transfers. In the clinical evaluation event, the CRS-R and GOS-E examinations item scores are collected. For the EEG evaluation event, the metadata associated to the recording settings for the resting-state, and task-based protocols (local-global and motor command protocol) are stored. The brain-body interactions event contains forms to collect metadata regarding the odor response and the 24hs respiration recordings. For the TMS-EEG event, the metadata associated with this procedure is stored. In the MRI event, forms to collect metadata related to fMRI during resting state and structural MRI were implemented. Information regarding the medication administered to the patient at the moment of each evaluation is also listed. As for the caregivers' events (C0, C1, C2, and C3) the items response for the above described subsets of questionnaires were stored. This longitudinal database, hosted at Paris Brain Insititute, unifies and centralizes the information collected by all partners ensuring data consistency and accessibility across centers.

### Participant recruitment and sample size

Two main groups of participants are enrolled for the PerBrain project: brain-injured DoC patients and their informal caregivers. Patients’ screening takes place at the Pitié-Salpêtrière Hospital, Paris (France), IRCCS Santa Maria Nascente Fondazione Don Carlo Gnocchi ONLUS, Milan (Italy), Loewenstein Hospital Rehabilitation Center, Raanana (Israel), University Hospital of the Ludwig-Maximilians-University, Munich (Germany), and Therapiezentrum Burgau Hospital for Neurorehabilitation, Burgau (Germany). Study inclusion takes place after the informed written consent of the patient’s legal guardian / next-of-kin has been obtained. After inclusion of a patient into the study, a patient’s relatives are invited to participate in the research as well. Participant recruitment started in September 2020 and data collection will last until at least April 2023. In total, 150 patients and 80 informal caregivers are expected to be enrolled.

### Eligibility criteria

#### DoC patients

DoC patients between 18 and 85 years are screened for study participation. Informed written consent must be provided by the patient’s legal guardian or, in case no legal guardian has been assigned yet, by the closest relative. As soon as a legal guardian has been appointed, he/she is asked to renew consent.

Exclusion criteria comprise pre-existing DoC, continuous medical sedation (induced coma), use of barbiturates for sedation, clinical instability, severe motor agitation, marked difficulty maintaining vigilance throughout the procedure, terminal malignant disease (as it increases the likelihood of not being alive for the 12-month follow-up assessment T3), prediction of highly unlikely survival until the time of the 12-month follow-up due to conditions such as multi-organ failure based on the judgment of the critical care physician, palliative care setting, withdrawal of life-support, and pregnancy.

For MRI and TMS-EEG assessments, the present study complies to specific contraindications (magnetic material in or on person such as pacemakers, defibrillators, intracranial metal implant, metal prostheses not compatible with MRI investigations). Formerly included DoC patients drop out of the study in case of palliative care setting or death.

#### Caregivers

Informal caregivers (spouse/partner, parents, children, siblings) of enrolled DoC patients are recruited for the caregiver study part. For each patient, only one relevant caregiver can be included in the study. Caregivers have to give informed written consent in order to participate, and must be between 18 to 85 years old. Exclusion criteria include: not knowledgeable about local language, severe current psychiatric comorbidity, and cognitive problems/neurodegenerative disease (inability to fill out questionnaires). Caregivers are free to drop out of the study any time they wish so; they anyway drop out from protocol in case of decease of their enrolled DoC patients.

### Measures and data analyses

#### qEEG

Quantitative EEG (qEEG) is recorded using locally available EEG systems (either 21 electrodes standard clinical systems or higher density systems with ≥ 32 electrodes, c.f. Table 3). For each patient, resting-state data as well as task-based data are collected at time points T1 and T2.

**Table 3:** Higher-density EEG recording systems used at the different study sites

Study site	System	Number of channels
Germany - Burgau	EGI400	256 electrodes
Germany - Munich	BrainVision	62 electrodes + 2 EOG channels
Israel	Biosemi	64 electrodes
Italy	BrainVision	60 electrodes + 2 EOG channels + 2 EKG channels
France	EGI300	256 electrodes

Resting-state recordings are run for at least 10 minutes continuously. Task-based conditions comprise two paradigms: a motor command protocol [31] and a local-global protocol (a two-level hierarchical oddball auditory protocol) [32]. For both protocols, stimuli are generated via a custom-built stimulation box (provided by the French project partner) which is connected to the specific recording EEG system of each site. Auditory stimuli (local-global paradigm) and pre-recorded standardized task instructions (motor command protocol) are presented to the patient via either headphones or loudspeakers at 70dB.

The motor command protocol consists of six block of eight trials alternating between the instructions “keep opening and closing your right/left hand” and “stop opening and closing your right/left hand”. The 10 seconds of EEG recording after the instructions were given are extracted and segmented in five epochs, each 2 seconds long. This procedure results in 480 epochs (5 epochs × 2 instructions × 8 trials × 6 blocks).

The local-global paradigm evaluates the processing of auditory irregularities [32]. For each trial 5 consecutive brief sounds (50 milliseconds duration) are presented with a 150-millisecond break between the sounds’ onsets and an inter-trial interval of 1350 - 1650 milliseconds. The fifth presented sound is either identical or different to the preceding four sounds, in that way defining the local level of auditory regularity. In addition, regularity can vary on a global level across trials (~80% frequent trials following regularity vs. ~20% violating regularity). In this way, two possible block conditions arise: BLOCK XX: with stimuli, local standard-global standard (frequent stimulus = 5 identical sounds), local deviant-global deviant (infrequent stimulus = 4 identical sounds followed by a 5th different sound), and BLOCK XY: with stimuli, local deviant-global standard (frequent stimulus = 4 identical sounds followed by a 5th different sound), and local standard-global deviant (infrequent stimulus = 5 identical sounds). During the patient assessments, every block condition consisting of 154 trials each is performed twice in a randomized order (1232 trials in total). Before the beginning of each block, patients are instructed to pay attention to the sounds.

EEG markers are extracted using the Python programming language. Pre-processing and computation of state-of-consciousness markers [26, 33, 34] use a designated software library (https://github.com/nice-tools/nice) built on top of the open source software libraries MNE [35]. Other indices quantifying simple and clinically relevant EEG features, such the EEG slowing, will be computed. Specifically, the spectral slope, which has been shown to accurately discriminate conscious from unconscious states in awake and anesthetized healthy subjects, will be estimated through a Matlab-based code that is freely available online [36].

#### TMS-EEG

TMS-evoked potentials (TEPs) are recorded using a TMS-compatible 64-channel Brainamp EEG amplifier (Brain Products GmbH, Germany) integrated with an MRI-guided neuronavigation system that ensures target reproducibility over subsequent sessions. Up to six cortical TMS targets are stimulated (one left and one right medial frontal sites, one left and one right medial parietal sites, one left and one right medial occipital sites) at different intensities spanning from 120 to 200 V/m [37–40]. During TMS-EEG recording, a customized noise [41] is played through in-ear earphones to mask the TMS click. Both single-trial and average TEPs are visually monitored in real time using the software rt-TEP [42] control for the possible contribution of artifacts and to maximize the impact of TMS on cortical neurons.

Following rejection of trials containing noise or muscle artifacts, EEG data are average referenced, high-pass filtered (1 Hz) and baseline corrected over 400 ms pre-stimulus. Independent Component Analysis (ICA) is applied to reduce ocular and residual muscle artifacts. Finally, data are low-pass filtered at 45 Hz. For each cortical target, a TMS-evoked response is obtained by averaging 150-250 artifact-free single trials. In order to obtain the overall amount of electrical activity induced by TMS, the Global Mean Field Power (GMFP) is computed from the multichannel average. Source modeling of TMS evoked potentials is performed only at the latencies corresponding to significant GMFP activations. To assess the threshold for significance, a bootstrap method (which does not assume normality) is applied by shuffling the time samples of pre-stimulus activity (from -300 to -50 ms) at the single-trial level and by calculating 500 surrogated pre-stimulus GMFPs. From each random realization, the maximum value across all latencies is selected to obtain a maximum distribution (control for type I error) and significance level is set at p<0.01. A similar procedure is applied to TMS-evoked responses recorded at single EEG channels. To evaluate the occurrence of pathological OFF-periods that may disrupt the emergence of global complex cortico-cortical interactions, time-frequency decomposition of TEPs is computed as described in [40]. Following theoretical considerations that link consciousness to the observation of complex EEG activity patterns that are integrated and differentiated at the same time, the Perturbational Complexity Index (PCI) [37, 43] is computed for each stimulation site and the individual maximum PCI value is used for diagnostic purposes. The prognostic contribution of all these neurophysiological measures is evaluated by comparing their longitudinal modulation with the clinical evolution of the patient. Notably, the tools and procedures described above will be shared by the Italian unit, which in the last two decades significantly contributed to the TMS-EEG field, with the German unit, the only other one provided with the proper equipment to carry out TMS-EEG measurements.

#### (f)MRI

For time points T1 and T2, patients undergo the following 3T MRI protocol with a 64-channel head/neck coil:

- T2-weighted and FLAIR-3D scans for lesion characterization and masking.
- High resolution 3D-T1-weighted images for the evaluation of the brain morphometric pattern.
- Diffusion-weighted images to evaluate the microstructure of the white matter, the main beams and the structural connectivity. Reverse phase encoding in a subsequent otherwise identical second run is applied to minimize susceptibility-induced distortions during post-processing.
- Susceptibility weighted imaging as an iron sensitive MR sequence to detect potential abnormalities like iron deposition in the brain, hemorrhages, micro bleeds and calcification.
- Resting-state fMRI sessions as well as their preceding field maps (phase and magnitude images) for functional connectivity analyses.

Prior to the preprocessing step, if possible, lesion maps are drawn using a semi-automatic software MRIcron (https://www.nitrc.org/projects/mricron/). The T1-weighted and functional scans are preprocessed using the fMRIprep package [44] (https://fmriprep.org/en/stable/index.html), which strives for robustness of preprocessing across different sites and scanners. fMRIPrep performs minimal preprocessing including motion correction, field unwarping, normalization, bias field correction, brain extraction, etc. Spatial smoothing is performed additionally, nuisance regression as well as normalization of the structural and functional data into a clinical template (mean age 65 years) [45]. The first five volumes of the functional scan are removed in order to ensure signal stability. Structural images are quantified using regional brain volumetry [46], functional images by quantifying functional networks [47], and dynamical interactions between brain areas [48].

#### Nasal airflow and respiration

A wearable recording device called the “Nasal Holter”, that was developed by the Israeli project partner, is used for assessment of nasal airflow and respiration. The body of the device contains batteries, a processor, two precise pressure sensors, on-board memory, and bluetooth, all encased in silicone. The device is ~7cm long, ~1.5cm wide, ~0.8cm thick, and weighs only ~10 grams. The holter is secured by a skin-safe sticker to the patient‘s chest or back of the neck, and connects to the nose using a custom nasal cannula with completely separate lines for left and right nostrils. Airflow in the nose generates a pressure wave through the cannula that is registered at the sensors. The processor then converts pressure back to air flow, that can be stored on the device or transmitted via Bluetooth. The device was tested and approved for safety by the Israeli Ministry of Health.

The device is used in two contexts: 1. Ongoing nasal respiration for 24 hours, with special attention to the nasal cycle, i.e., the left/right asymmetry in nasal airflow that is indicative of autonomic tone [49], and 2. The "sniff-response", i.e., an automatic modulation in nasal airflow in accordance with odorant properties [50]. Both the 24-hour recording and odor experiments are initiated via a cellphone-based application.

For the 24-hour nasal airflow recording, the nasal cycle is assessed by the Laterality Index (LI). This index measures the flow ratio between the left and right nostrils, calculated using: $\textrm{LI}=\frac{\left({\textrm{Flow}}_R-{\textrm{Flow}}_L\right)}{\left({\textrm{Flow}}_R+{\textrm{Flow}}_L\right)}$ for every minute. The result of this calculation is a one-minute resolution time series of lateralization extent, with a value of 1 representing airflow through the right nostril only, a value of -1 representing airflow through the left nostril only, and a value of 0 representing equal flow across nostrils [49]. Healthy adults have a nasal-cycle shift every ~2 hours during wake, and ~4.5 hours in sleep. Frequency of shifting is expected to diminish as a function of severity of DoC. Slightly reduced shifting in MCS, and no, or near-no shifting in UWS are hypothesized.

In the odor experiment, at each trial, jars containing a pleasant odorant, unpleasant odorant, or blank stimulus are presented to the patient. An audible voice instructs the patient to "please prepare to sniff the odor at the tone, three, two, one” followed by a 1-second beep-tone. The experimenter brings the jar to under the patient‘s nose at the "beep" count, and removes it after five seconds. This procedure is repeated 30 times (10 times for each odor stimulus, random order), with ~30s ISI, resulting in a total experiment duration of ~15 minutes. Using this data, we test for a sniff-response, namely a modulation in nasal airflow in accordance with the odorant. A sniff-response is defined as a trial meeting at least one of the the two following criteria: 1) an odorant-induced reduction in sniff magnitude of 15% or more in relation to baseline respiration. 2) a air-flow standad deviation across all trials in the session of more than 0.35. Meeting either of these criteria implies MCS rather than UWS [50]. These two hypotheses, namely a nasal-cycle and sniff-response reflecting levels of consciousness, may enable the addition of a simple tool to the arsenal of instruments used in assesement of DoC.

#### Multilayer integration of multimodal data

The data from the patients’ multimodal assessments collected at the different international study sites are fed into a central analysis unit for the overall project that is maintained by the French project partner. Multivariate procedures to integrate information across the different modalities of the hierarchical evaluation are developed. Previous results indicate that integrating information from different markers within a given modality of evaluation (i.e using EEG or fMRI) can boost diagnostic performance [33, 34, 47]. This logic is extended to integrate information across evaluation modalities and layers within the modalities (e.g. the three different paradigms of the EEG modality).

A critical aim of the PerBrain project is to evaluate if integrating information from different modalities in the hierarchical evaluation conveys additional diagnostic evidence. To do so, classification efficiency of the markers extracted from the different multimodal modalities and layers will be compared and obtained multimodal information will be integrated into an optimal, multimodal consciousness diagnosis. For the development of the decoder the expertise demonstrated in earlier research on signatures of consciousness [33, 34] will be followed and different multivariate integration strategies will be applied (such as mass-univariate analysis and training of support vector machines, random forest classifiers or other machine learning algorithms). This procedure will be evaluated using cross-validation techniques and generalization across the datasets in the different recording sites. Machine learning procedures will be implemented in Python using scikit-learn [51].

#### Caregivers’ quantitative questionnaires and qualitative interview

Similar to the patient assessments, the well-being of the patients’ caregivers is tracked for a period of 12 months using a battery of quantitative questionnaires. Data are collected at four time points (C0-C3; see Table 1): before patient’s T1 multimodal assessment, subsequently 1 - 2 weeks after results communication of the performed multimodal assessment at T1 and T2, and after patient’s behavioral assessment at T3. Questionnaires include queries about sociodemographic characteristics (e.g. age, gender, relation to patient, education), religion and spirituality, as well as scales measuring perceived fear and burden due to current COVID-19 situation (Fear of COVID-19 Scale) [52], cognitive and emotional representations of the patient’s illness (Brief Illness Perception Questionnaire) [53], perceived personal quality of life in general (EQ-5D-5L) [54] and as a caregiver (Adult Carer Quality of Life Questionnaire ACQoL) [55], anxiety and depression levels (Hospital Anxiety and Depression Scale) [56], degree of resilience (Resilience Scale 14) [57], sense of coherence (Sense of Coherence Scale 13) [58], and attitudes towards surrogate treatment decisions (Treatment choices survey) [59]. Questionnaires are filled in together with an examiner, either in person, through video conferences, or during a telephone interview.

In addition to the administration of quantitative questionnaires, a subset of caregivers (N = 9) are interviewed using a semi-structured grid that was developed by a sub-group of researchers within the project team together with two graduate students prior to recruitment. The interviews are conducted after the notification of thedisclosure of the results of findings stemming from the multimodal consciousness diagnostics at C1. The aim is to gain new insights into the specific and individual needs of the caregivers concerning communication with the medical staff regarding the surrogate treatment decisions for the patients, their ethical implications as well as caregivers’ epistemic believes about the nature of knowledge about the patient's condition. Caregivers are enrolled for qualitative interviews according to the principles of consecutive purposive and maximum variation sampling.

For analyses of quantitative caregivers’ data, descriptive, correlational and predictive statistics are calculated for the four time points and across points. The qualitative caregiver interviews are transcribed and translated into English. Reflexive thematic analysis [60] is performed on the translated interviews to identify caregivers’ needs, expectations, and their underlying assumptions and attittudes regarding clinical communication of technology-based results of the performed neurodiagnostics. The gained knowledge is used for the development of an information brochure for caregivers of DoC patients to facilitate the communication between them and health professionals. The brochure development follows the framework by [61]. It is supervised by a steering group of experts of different disciplines and with various (professional) backgrounds (e.g. counseling, medical ethics, neuropsychology, or health communication, and engaged caregivers) and it will be tested by family caregivers.

## Discussion

DoC patients are characterized by extreme disability as well as variability concerning clinical phenotype, associated (neurological) deficits and underlying brain pathologies [1, 62]. The diagnosis, prognosis, and resulting daily care of this group of neurological patients pose major clinical as well as social challenges. Due to the application of non-standardized clinical routine examinations and diagnostic tests requiring active behavioral participation of the examined patient, a high rate of DoC patients are erroneously deemed unconscious [5]. Misdiagnosis can lead to prognostic mistakes, ineffective rehabilitative approaches, and psychological stress of the caregivers. Linked to that are immense personal and medical ethical concerns as well as high economic costs. Diagnosis has a direct impact on major clinical decisions regarding rehabilitation or life-sustaining therapy [63]. Underestimation of remaining consciousness levels might result in therapeutic nihilism, withdrawal of care, or withholding of neuro-intensive or neuro-rehabilitation treatment. Therefore, it is of utter importance for both affected patients and their family members to improve diagnostic and prognostic accuracy and to identify reliable markers of consciousness.

An extensive body of research suggests that a parallel multimodal assessment of DoC patients leveraging different neuroimaging and neurophysiological techniques can enhance diagnostic accuracy, detection of covert consciousness, and prognostic reliability [12, 15, 64–66]. The multicentric, longitudinal PerBrain project aims at applying modern multimodal techniques to improve the individual DoC diagnosis and outcome prediction. This is supposed to be achieved by developing patient-tailored and individualized diagnostic hierarchical pathways guided by behavior and innovative multimodal techniques, which can ultimately lead to individualized therapy planning.

Due to the heterogeneity of this specific clinical condition, large patient samples are required to train and test diagnostic techniques among all possible clinical conditions. To that end, clinical partners from four different countries (France, Italy, Israel, and Germany) are cooperating in the PerBrain project, each contributing expert knowledge and experience regarding different aspects of DoC and its investigation. As a further key advantage of the PerBrain project, the presented multimodal approach allows assessing DoC patients even if they have contraindications to one of the applied neurodiagnostic techniques but not the others (e.g. MRI contraindication). In that way, a wide range of the heterogeneous group of DoC patients are eligible for study participation and inherent drawbacks of the distinct techniques can be compensated. To account for replicability and widespread clinical applicability, the developed analysis algorithms will be made publicly available at the end of the project for automated computation of patient-specific evaluations in other clinical centers. Therefore, the PerBrain project makes a decisive contribution to personalized medicine in this severe and particularly challenging clinical condition.

In addition, PerBrain seeks to improve the communication of technology-based results with the patients’ informal caregivers. Facing a DoC diagnosis and an uncertain outcome prediction poses a highly stressful situation for informal caregivers, which can lead to severe mental and physical health issues [25–27]. In the present project, factors that affect well-being of and treatment decision-making by informal caregivers/family members confronted with multimodal technology-based tests for DoC patients are investigated across different countries with different social cultures in order to develop optimal communication strategies in clinical practice for effective communication of technology-based results.

## Data Availability

The sharing of raw data will be restricted to the partners of this project. In order to protect the anonymity of patients, consent forms do not include a possibility for sharing the raw data with researchers outside the project. Final processed data will be publicly deposited at the time of publication of associated manuscripts, to extend the lifetime of all data output beyond the end of the project with an exception to the context-rich qualitative caregiver data. Scripts used to process data will be published on GitHub at the end of the project.

## Abbreviations

AcQoL: Adult Carer Quality of Life Questionnaire
BIPQ: Brief Illness Perception Questionnaire
CMD: Cognitive Motor Dissociation
CRS-R: Coma Recovery Scale Revised
DoC: Disorders of Consciousness
EQ-5D-5L: European Quality of Life-5 Dimensions
FCV-19S: Fear of COVID-19 Scale
fMRI: Functional Magnetic Resonance Imaging
GMFP: Global Mean Field Power
GOS-E: Glasgow Outcome Scale Extended
HADS: Hospital Anxiety and Depression Scale
ICA: Independent Component Analysis
LI: Lateralization Index
M: Months
MCS: Minimally Conscious State
MRI: Magnetic Resonance Imaging
PCI: Perturbational Complexity Index
qEEG: Quantitative Electroencephalography
RS14: 14-Item Resilience Scale
rsfMRI: Resting-state fMRI
SOC-13: 13-item Sense of Coherence Scale
SWI: Susceptibility Weighted Imaging
TEPs: TMS-evoked Potentials
TMS-EEG: Combined Transcranial Magnetic Stimulation and Electroencephalography
UWS: Unresponsive Wakefulness Syndrome
VS: Vegetative State
W: Weeks
